# Flow Cytometry: A Rapid and Robust Adjuvant Technique for Pathological Diagnosis

**DOI:** 10.5505/tjh.2012.62533

**Published:** 2012-03-05

**Authors:** Monica Jain, Anil Handoo, Dharma R Choudhary, Atul Bhasin

**Affiliations:** 1 Dr B L Kapur Memorial Hospital, Department of Hematology, New Delhi, India; 2 Dr B L Kapur Memorial Hospital, Department of Clinical Hematology, New Delhi, India; 3 Dr B L Kapur Memorial Hospital, Department of Internal Medicine, New Delhi, India

## TO THE EDITOR

Flow cytometry is a powerful technique for correlating multiple characteristics of single cells. A series of 2 cases are described to illustrate the pivotal role of this technique in routine hematological diagnosis.

## CASE 1

An 18-year-old male presented with a 10-d history of fever, sore throat, and hepatospleenomegaly. CBC revealed leukocytosis (19,000 mm–^3^), 71% atypical lymphoid cells with nuclei showing opened-up chromatin and a moderate quantity of cytoplasm mimicking lymphoblast/lymphoma cells, and an Hb of 11g dL–^1^ and PLT of 145,000 mm–^3^.

Immunophenotyping was performed using CD45, CD19, CD3, CD5, CD7, CD4, CD8, CD38, CD56, kappa, and lambda light chains. Gating was performed using forward- side scatter and CD45-side scatter.

A single large cluster observed in the lymphocyte region of the SSC/CD45 side scatter showed a high level of CD45 expression and was composed predominantly of T-cells (CD3+) with moderately high CD38 positivity (T-cell activation marker). Nothing abnormal with respect to T-cell markers (CD3/CD5/CD7) was noted. Reversal of the CD4:CD8 ratio (0.1) was also noted. CD19, kappa, lambda, and CD56 expression was not observed. Immunophenotyping results suggested non-neoplastic proliferation of lymphocytes—primarily involving a T-cell component with an inverted CD4:CD8 ratio, which could also be a manifestation of immunosuppressive states and viral infections, e.g. EBV, CMV , and HIV [1]. EBV IgM was positive (17.58 U mL^-1^) (normal: < 8.00 U mL^-1^) and HIV antibody testing was negative; thus, the diagnosis of infectious mononucleosis was confirmed.

## CASE 2

A 57-year-old female that was followed-up for multiple myeloma presented with severe pain around the rib cage. CBC showed hemoglobin-7.2g/dL and platelets-80 X 10^3^/ μL with an occasional atypical lymphoid cell. Bone marrow aspirate was hemodilute, and showed large bizarre cells with prominent nucleoli. Immunophenotyping of bone marrow aspirate was performed using CD45, CD19, CD56, and CD38 (a limited panel of markers was used due to financial constraints). Gating was performed using forward-side scatter, CD45-side scatter, and CD19-SSC. Bone marrow showed a cell cluster comprising ~15% events in monocyte region of the FSC/SSC dot plot. A CD45/SSC dot plot showed dim CD45 expression with moderate SSC expression; the cells were CD19 negative, and exhibited high-level CD38 and variable CD56 expression. The morphology and immunophenotypic profile were consistent with relapsed multiple myeloma.

Flow cytometric immunophenotyping remains an indispensable tool for the diagnosis, classification, staging, and monitoring of hematologic neoplasms. It is a rapid and robust adjuvant technique for the diagnosis of hematolymphoid neoplasms, and can be easily performed using peripheral blood or bone marrow aspirate material for diagnostic confirmation. Reactive lymphocytosis is common in children with viral infections; however, when circulating lymphocytes are large and have atypical features their immature and uniform appearance indicate the possibility of a hematolymphoid neoplasm. In such instances peripheral blood flow cytometry is an important tool for excluding neoplastic pathology without subjecting patients to the procedure of Bone marrow. Aspiration/biopsy.

Research has shown that acute infectious mononucleosis is characterized by an activated CD8+ T-cell population and antigenic down regulation of CD7, and or CD5, in addition to a decrease in B-cells and the CD4:CD8 ratio [2]. Case 1 had ≈94% T-cells with a CD4:CD8 ratio of 0.1; however, nothing abnormal related to CD5 or CD7 was noted. Most cases of plasma cell dyscrasia are diagnosed without Flow cytometry; however, it may be useful in certain patients with hematologic abnormalities and an elevated level of plasma cells in the bone marrow and without specific clinical manifestations [3]. Flow cytometry may also be helpful in the differential diagnosis of unusual cases of Multiple Myeloma [4].

Case 2 had been followed-up for multiple myeloma and the bone marrow aspirate exhibited abnormal bizarre lymphoplasmacytic cells. Immunofixation electrophoresis showed only a faint band in the early gamma-region. Normal immunoglobulins were also noted in the IgG and kappa lanes; therefore, flow cytometry was performed in order to rule out any other secondary malignancy [5]. Normal plasma cells are polyclonal and exhibit high-level CD38 and CD19, and variable CD45 expression, but no CD56 expression. In contrast, malignant plasma cells are monoclonal and exhibit lower levels of CD38 expression; CD45 is generally expressed at a low level, and CD19 is undetectable, whereas CD56 expression is high [6]. CD38 is a non-specific marker that can be observed on activated T-cells and B-cells. CD138 gating identifies a much more homogeneous population of myeloma cells than does CD38 gating. Thus, the combination of CD38 and CD138 is superior to CD38 alone for identifying CD45+ myeloma [7]. Due to financial constraints a single tube with 4 markers was used in case 2, which showed low-level CD45, high-level CD38, variable CD56, and no CD19 expression. In conclusion, flow cytometric immunophenotyping is a powerful and rapid technique for correlating multiple phenotypic characteristics of single cells.

## CONFLICT OF INTEREST STATEMENT

The authors of this paper have no conflicts of interest, including specific financial interests, relationships, and/ or affiliations relevant to the subject matter or materials included.

## Figures and Tables

**Figure 1:**
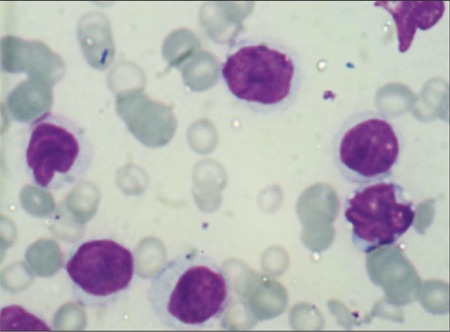
Morphology of cells.
